# Electrical Impedance Tomography – Recent Applications and Developments

**DOI:** 10.2478/joeb-2021-0007

**Published:** 2021-11-20

**Authors:** Sofiene Mansouri, Yousef Alharbi, Fatma Haddad, Souhir Chabcoub, Anwar Alshrouf, Amr A. Abd-Elghany

**Affiliations:** 1 Department of Biomedical Technology, College of Applied Medical Sciences, Prince Sattam bin Abdulaziz University, Al-Kharj, Saudi Arabia; 2 Laboratory of Biophysics and Medical Technologies, Higher Institute of Medical Technologies of Tunis, University of Tunis El Manar, Tunis Tunisia; 3 Biophysics Department, Faculty of Science, Cairo University, Cairo Egypt

**Keywords:** Electrical impedance tomography, instrumentation, image reconstruction, medical applications, imaging technique

## Abstract

Electrical impedance tomography (EIT) is a low-cost noninvasive imaging method. The main purpose of this paper is to highlight the main aspects of the EIT method and to review the recent advances and developments. The advances in instrumentation and in the different image reconstruction methods and systems are demonstrated in this review. The main applications of the EIT are presented and a special attention made to the papers published during the last years (from 2015 until 2020). The advantages and limitations of EIT are also presented. In conclusion, EIT is a promising imaging approach with a strong potential that has a large margin of progression before reaching the maturity phase.

## Introduction

Medical imaging has become essential since it allows the physiology and anatomy of the human body to be indirectly visualized, thus offering doctors the possibility of making increasingly precise diagnoses. Its evolution was spectacular. Wilhelm Röntgen, while working on cathode rays happened to discover x-rays by chance in 1895 [1]. It was the decisive step for medical imaging. Mamography [2], CT scan [3, 4, 5], Nuclear Medicine [6, 7, 8], Ultrasound [9], positron emission tomography (PET) [10], single-photon emission computerized tomography (SPECT) [11], magnetic resonance imaging (MRI) [12] are imaging techniques and an undeniable investigative tool for visualizing biological processes. They are essential for understanding physiology and pathologies in order to achieve better diagnose, prognosticate and eventually treat them. Each of these imaging modalities have unavoidable advantages and disadvantages that affect both the patient and the healthcare professional.

Electrical impedance tomography (EIT) is an imaging technique that reconstructs images of a specific region in the human body based on the electrical conductivity of biological tissue. Following the impedance camera designed by Henderson and Webster [13], Barber and Brown suggested the first electrical impedance tomography system that produced a tomographic image based on electrical resistivity [14]. Eventual research led to the design of a new system called Sheffield by Brown and Seagar, which has been widely used until now [15].

In this paper, we mainly focus on EIT imaging technique and its applications. Although EIT is still in its infancy and not yet considered a routine examination, it is promised a boom. Compared to techniques such as ultrasound or X-rays, EIT permits to assess the physiology and potential pathological evolutions, such as the evolution of a tumor. It is a not harmful, but a noninvasive and real time technique. In addition, its reduced cost would increase its use, Table 1.

**Table 1:** Comparison between different imaging modalities.

Medical imaging modality	CT	US	MRI	EIT
**Basic principle**	X-rays	High frequency sound	Radio waves	Impedance
**Types of radiation**	Ionizing radiation	Non-Ionizing radiation	Non-Ionizing radiation	Non-Ionizing radiation
**Contrast**	High	Low	High	Low
**Spatial Resolution**	50-200 μm	50-500 μm	25-100 μm	Low
**Scanning time**	<20 min	< 30 min	<40 min	<10 min
**Cost**	Moderate	Low	Very High	Low
**Size**	Non portable	Portable	Non portable	Portable
**Advantages**	Bone and tumor imaging, anatomic imaging	Visualize muscles, tendon and internal organs	Morphological and functional imaging	Rapid tomographic imaging, low cost, noninvasive
**Disadvantages**	High cost Ionizing radiation	Operator dependency	Noisy, cost, low sensitivity	Not mature yet

**Table 2:** Electrical conductivity for Human tissues.

Tissue	Conductivity (*mS/m*)
Cerebrospinal fluid	1450 - 1800
Blood	500 - 650
Scalp	300 - 400
Brain	300 - 420
Muscle	200 - 400
Fat	50
Bone	6

**Fig.1:**
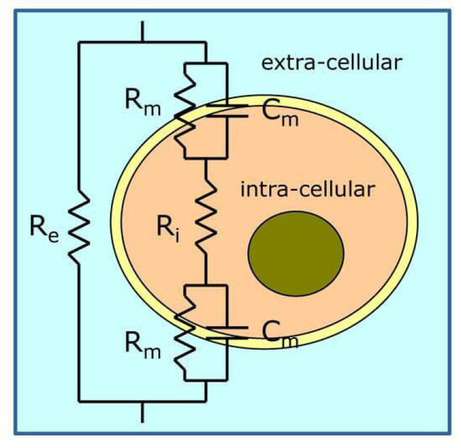
The equivalent electrical circuit model for tissues.

Biological tissue is made up of multiple cells with conductive fluid surrounded by an insulating membrane. The latter is at the origin of the electrical properties of cells. It is made up of phospholipids and proteins [16]. Webster proposed a simple electric model representing the cell [17], Fig. 1.

The membrane is assimilated to the structure of a membrane capacitor (Cm) in parallel with a membrane resistance (Rm). The extracellular and intracellular fluids are modeled respectively by the extracellular (Re) and intracellular (Ri) resistances. The value of Rm is generally much larger than Ri and Re. If a low frequency current is applied, the current cannot pass through the membranes, so the impedance of the cell membrane is relatively high at low frequencies. In a set of cells, the electric current then tends to take the path of lower impedance, that is to say the extracellular fluid, [18]. At high frequencies, the capacitive component (Cm) of the cell membrane will short-circuit the resistance (Rm) of this membrane. The current will then be able to pass more easily through the cell membrane and the overall conductivity of the tissues will be higher. This model is too simple, because an actual tissue sample would be represented as a vast network of interconnected modules of this form.

Since the 1900s, researchers have shown interest in measuring the electrical impedance of biological tissues. Galeotti was the first to note the changes in resistivity that accompanies cell death [19]. In freshly excised tissue, he observed an initial increase in electrical resistance followed by a dramatic decrease in resistivity. In 1967, Geddes and Baker demonstrated the specific resistance of biological tissues, including heart muscle, body fluids, skeletal muscle, blood, liver, lung, kidney, pancreas, nervous tissue, fat and bones [20]. Additionally, they revealed that the electrical properties of malignant tissue are significantly different from those of healthy tissue. The measurement of electrical impedance of tissues or cells is given by the following expression (Equation (1)):


(1) Z = R + jX 

knowing that the electrical impedance, Z, is a complex number. R and X represent the electrical resistance and reactance, respectively.

It can be seen that the electrical impedance Z is an integral measurement which is of no interest in imaging. In electrical impedance imaging, an image of the ability to conduct an electrical current should be measured for the material inside an object. Thus, the electrical properties measured in electrical impedance imaging are conductivity σ and permittivity ε [21].

Conductivity is expressed in siemens per meter (S/m) (see Equation (2)), where J is the current density at a given location in a resistive material, E is the electric field at that location, and σ is a material-dependent parameter called the conductivity [22].


(2) σ=J/E

The macroscopic values of permittivity and conductivity available in the literature show that biological tissues have unique material properties [23]. Conductivity and permit-tivity vary at certain applied frequencies. The permittivity values are very high at the lowest frequencies. Consequently, soft tissue is a good conductor and exhibits high electrical conductivity. In addition, adipose tissue has less conductive properties (0.08 S/m) in comparison with muscle tissue (0.5 S/m), which is about ten times the former tissue [24].

The average conductivity of some tissues varies for a variety of reasons. In the case of the lungs, conductivity depends on the volume of air they contain, the air being poorly conductive. In the case of muscles, the conductivity depends on the direction of the muscle fibers; it is lower in the transverse direction and higher in the longitudinal direction. It is noted that these values cover approximately three orders of magnitude. Tissue types could therefore be discerned if absolute conductivity measurements could be made with sufficient precision. Table 2 shows the average values of electrical conductivity for different tissues at the excitation frequencies commonly used in EIT [25].

## Electrical impedance tomography

There are three types of electrical impedance tomography; conventional EIT, dual frequency and multi-frequency EIT. The conventional EIT uses a single frequency, most often 50 kHz. Images are constructed from two measurements at two different times to observe the change in conductivity. The images obtained, representing a change in conductivity in the time domain, are called differential images. This image shows the evolution of tissue resistivity between the reference and data points. Differential imaging avoids the problem of having to take into account the 3D shape of the body to be imaged [14]. The latter imaging technique minimizes the effect of instrumentation errors since it is presented in both sets of measurements. However, it associates limits linked to the stability of the reference which is not guaranteed for long-term acquisitions [21, 26].

Dual-frequency EIT (EIT-DF) illustrates changes in conductivity as a function of frequency. EIT-DF applies currents at two different frequencies [21], and performs measurements at two frequencies that allow differentiation between two types of tissue, thus revealing abnormalities in their cellular structure. This technique minimizes the reference stability errors by using two frequencies simultaneously.

In Multi-frequency EIT (EIT-MF) systems, each biological tissue has a specific conductivity that varies with frequency. This type of tomography consists in applying sinusoidal currents at different frequencies, either sequentially or simultaneously by the superposition of sinusoids at several frequencies. Recently, several EIT-MF systems have been developed to image physiological processes that evolve slowly over time [21, 27].

### Instrumentation

In different EIT systems, voltage can be measured by applying a current ranging from 1 kHz to 1 MHz to an object by connecting two electrodes to the object surface [23].

**Fig. 2:**
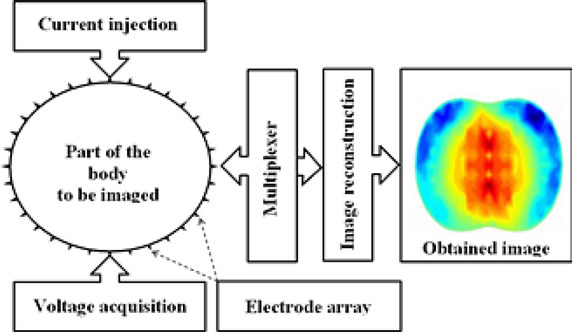
Main parts of an EIT imaging system.

The image reconstruction depends on the number of electrodes used. The system can have 8, 16, 32, 64, or even 128 electrodes. However, due to space and the limitation of data collection, it is difficult to have more than 256 electrodes in an EIT system. Thus, the number of independent measurements is limited and therefore the resolution is low. Usually, the electrodes are evenly spaced and surround the object surface that will be imaged. Although adjacent simulation and measurements are not always used and in some respects not recommended [27], a sinusoidal current is injected between two adjacent electrodes, and as a consequence, the potential difference is acquired at all other pairs of adjacent electrodes. Such a set of measurements is called a projection. In addition, it can be easily distinguished between serial, parallel and semi-parallel data acquisition systems. Serial data acquisition systems are composed with a single current source and a single measurement chain. Parallel data acquisition systems are composed with N current sources and N measurement chains. Semi-parallel data acquisition systems with a single current source and N measurement channels, [28]. A basic EIT system is composed by a current injector block, to inject the desired current through the electrodes to the concerned zone. A conditioning signal block processes the voltage signals developed on the sensing electrodes and transfer them to the data acquisition system. A multiplexer module is used to select the adequate injection or detection electrodes [28, 29]. Furthermore, a computer with an image reconstruction algorithm [30, 31] and a set of electrodes [32, 33] attached to the test subject to inject currents and measure voltages, Fig. 2.

### Image reconstruction

The EIT reconstruction process involves three components: the data, the main model (geometry, position of the electrodes) and the reconstruction method (forward or inverse problem). The reconstruction of the electrical properties by the forward problem consists in constructing a digital model of the area to be imaged capable of determining the potentials and currents expected at the level of the electrodes for a given distribution of electrical properties. The reconstruction by the inverse problem estimates the electrical properties of the imaged object, via an optimization function which adjusts the distribution of electrical properties to minimize the difference between the values calculated by the forward model and the experimental measurements. The aim of the inverse problem is to obtain the conductivity distribution using the boundary voltage vector and the injected current vector [34, 38].

The measured voltages are a function of four parameters: the position and shape of the electrodes, the intensity of the currents applied, the conductivity distribution of the part of the body to be imaged, and the shape of this part. From all the measurements, an image of the electrical conductivity can be reconstructed. Two types of images can be obtained. Static images represent the distribution of conductivity at a given time without using reference data. The reconstruction algorithms therefore use a single series of measurements. Dynamic images represent the variation of the conductivity distribution between two instants. The reconstruction algorithms use two series of measurements [35]. Data can be acquired at different times (DTEIT) or frequencies (DFDEIT). DTEIT produces images between two different time intervals of a region variation in the conductivity distribution [17, 39], while DFEIT reconstructs the impedance distribution from two discrete frequencies [40, 41].

**Fig. 3:**
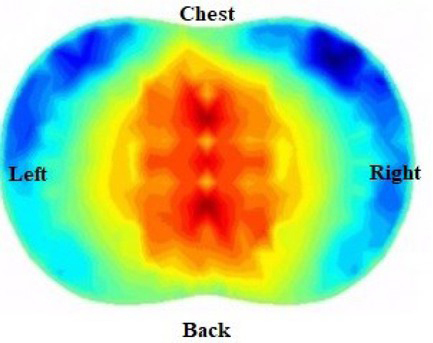
Chest image reconstruction by EIT [21].

EIT generates cross-sectional images of the internal distribution of electrical impedance continuously at the bedside, which explains its high temporal resolution [42, 43]. Poor posture means the solution for intrathoracic impedances may be unstable: small errors in voltage measurements disrupt reconstruction. This is related to the size and number of electrodes. In order to overcome this problem, most algorithms use assumptions called regularizations, such as the smoothness of the distribution of intrathoracic impedance [26, 44]. These regularizations help the algorithm to choose between competing solutions, producing a reasonable picture of the true distribution of impedance in the thorax, at the expense of degraded spatial resolution. Fig. 3 presents a chest image reconstruction by EIT.

To obtain images of the conductivity distribution, various reconstruction algorithms have been used.

### Linear approach to image reconstruction

The most precise approach is based on the following algorithm: a transformation parameter (Ac) is set up to determine the voltage, V, produced from the conductivity distribution, c, (see Equation (3)). Ac is a function of c, so it is necessary to assume an initial distribution, c_0_. The expected V is calculated and compared to the real measured voltages, V_m_. It can be shown that an improved estimate of c is given by (4):


(3) V = Ac×c

(4) $$C_{1}=C_{0}+\Delta C$$

The improved c value is then used in the iteration to calculate an improved estimate of V. This iterative process is continued until some appropriate limit point is reached. In the presence of noise measurement, the iteration is stopped when the difference between V and V_m_ is within the margin of error generated by a known noise on the data. There are some practical difficulties associated with this approach: a large variation of c cannot produce small variations of V [45].

### Single-step reconstruction

The complete reconstruction problem is nonlinear and requires iteration. However, each step of the iterative process is linear. Images are reconstructed using only the first iteration step, a roughly justified assumption for small changes in conductivity from a uniform. Most of clinical images produced to date have used a one-step reconstruction algorithm. Although this algorithm uses very suboptimal current models, which has not yet been a limitation due to the high quality of the data collected and the limited number of electrodes used [46].

### Sheffield back-projection

The back-projection algorithm, developed by Barber and Brown in 1983, is an efficient algorithm with a relatively low computational cost [47]. This algorithm is based on the same principle of computed tomography: it superimposes the 16 voltage profiles on top of each other. Reconstruction artifacts are removed by applying selective boundary filtering. The resulting image displays the region of increased impedance (E) at the correct location [48]. The projection of the displayed EIT images is cranial caudal as with scanners. This means that the left side of the image displays the right side of the patient. The upper part of the image shows the ventral aspect of the patient. However, this Sheffield back-projection reconstruction algorithm has some inherent limitations, such as fixed geometry to round-shaped subjects, no way to remove corrupted data, and no flexibility regarding the sequence of current injections and voltage measurements. Thus, it has been shown that iterative approximation methods, such as the Newton-Raphson algorithms, give better results.

### Newton-Raphson

**Fig. 4:**
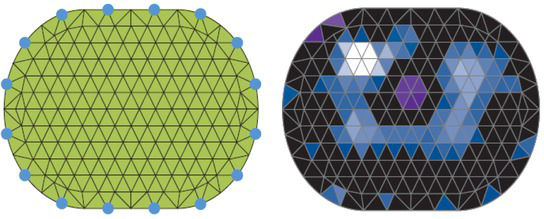
FEM reconstruction, Left: 2D mesh with 340 finite elements for 16 surface electrodes. Right: FEM-based reconstruction of the bioimpedance distribution.

The linear Newton-Raphson reconstruction algorithm is based on the Finite Element Method (FEM) to convert measured voltages to an EIT image. This algorithm is more specific to lung 2D imaging. The method divides the surface in the electrode plane into several triangular elements,

where each element has homogeneous bioelectric properties. This method allows the calculation of the resulting surface tensions at the limit nodes of the model, for any arbitrary distribution of the impedance values within the mesh, which is the numerical solution of the forward problem. For the reconstruction of EIT images, after measuring the tensions on the body surface their relative changes are introduced into the Newton-Raphson reconstruction algorithm by multiplying them with a sensitivity matrix. Recently, this matrix has been optimized by taking into account the actual EIT data from several hundred patients [48]. The Newton-Raphson algorithm attempts to assign a change in relative impedance to each individual finite element to get a better match for the numerical solution of this "inverse problem" from FEM to the actual voltage profile. After reconstruction of the image, the triangular structure is converted into a rectangular pattern for further image processing. In addition, the damping of the boundary artifacts is applied, Fig. 4.

### Optimization by particle swarms

**Fig. 5:**
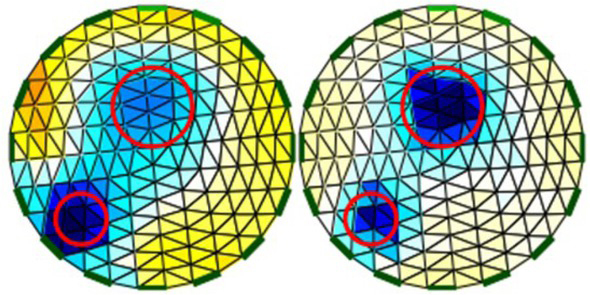
Comparison of the phantom reconstruction obtained with the linear inverse solver (left), and the proposed method using ANN and PSO (right). Both targets are indicated by red circles [54].

Newton's algorithm, which is used to solve the inverse problem, is a very sensitive to the initial values. Thus, to overcome this drawback, Particulate Swarm Optimization (PSO) can be used [49]. A conductivity-based clustering algorithm, a modified Newton-Raphson algorithm, can also be combined with an adaptive PSO algorithm to improve the quality of the reconstructed image. Compared to a Newton-Raphson algorithm, this method converges faster to an optimal solution and has a higher spatial resolution on a reconstructed image [50, 51]. One can also combine the PSO and the Gauss-Newton regularization algorithm (PSO-tGN), in which the PSO algorithm is used to obtain the initial electrical impedance distribution. The Gauss-Newton regularization algorithm is used to solve the problem iteratively. We can also combine PSO with the artificial neural network (ANN) method to solve the inverse problem of EIT [52]. This method gives a nonlinear conductivity distribution, which greatly reduces the presence of artifacts in the final image [53]. The nonlinear method is able to solve the inverse problem with high precision and works better than the classical linear inverse solver, Fig. 5.

### 3D EIT

Two-dimensional EIT (EIT-2D) reconstructs the spatial distribution of the impedance profile from current-voltage measurements developed by applying sinusoidal constant current through the skin electrodes fixed in a 2D plane in the body of the patient [17]. Thus, the EIT-2D approximately reconstructs the 2D conductivity distribution assuming that the electric current is conducted in a 2D plane. As the electric current is not confined in the plane of the array of electrodes, but rather spreads over a 3D space inside the volume, the EIT-2D generates errors brought by this 3D conduction of the electric current [17]. In addition, the EIT-2D of a considered tissue provides only the 2D impedance distribution. No information about the 3D anatomy and physiology. 3D EIT provides a 3D conductivity distribution with better visualization of tissue interior, which helps physicians and clinicians better characterize tissue [17].

### Graz for 3D-EIT (GREIT)

The Graz Reconstruction Algorithm for EIT (GREIT) is more adapted to the pulmonary EIT [55]. This algorithm is initially limited to cylindrical geometries with a planar electrode arrangement. Subsequent adaptations allowed reconstructions on an arbitrary geometry [56]. Q. Li et al. proposed and tested in a micro-EIT with 360 electrodes, an extension of the GREIT algorithm in 3D [57]. A similar implementation using a commercially available 32-electrode EIT system for lung imaging [58] has shown that it is possible to extend the application of this popular pulmonary EIT algorithm to other areas, such as imaging of the head or breast. For the reconstructed image, each row corresponds to a layer of voxels and each column to a different target position.

Several other methods or approaches and in particular DBAR methods can allow a novel pathway in comparison with traditional or regularized linear methods [59]. Likewise, the increased use of deep learning will greatly influence the reconstruction methods in the future [60, 61, 62]. Table 3 summarize the main image reconstruction algorithms.

**Table 3:** Image reconstruction algorithms.

Reconstruction algorithm	Description
Linear approach	- Solves the forward problem - Iterations are limited
Simple stage reconstruction	- Solves the forward problem - Iterative processm - Very much used
Sheffield Back-projection	- Solves the forward problem - The best known for EITm - Not flexible
Newton-Raphson	- Solves the forward problem based on EMFm - Inverse problem thanks to matrix sensitivity
Optimization by particle swarms	- Solves the inverse problem

## Applications of EIT

EIT presents different advantages. It is a non-invasive technique, radiation-less, low cost, safe and easy to use and suitable for bedside measurement and intensive care unit monitoring. Currently, significant research and development work is being done in the study of this promising technique thanks to these advantages [63]. This section is devoted to the description of these different medical applications.

### Monitoring of brain activity

EIT has the potential to image brain activity, [64, 65, 66, 67]. There are two mechanisms that lead to changes in impedance during normal brain activity, [68]. *Slow changes* activities occur due to the increase in cerebral blood volume during metabolic activity, over tens of seconds. These resistive changes in the brain can be detected as voltage changes on its surface, and because the injected current is known, these resistive changes can be reconstructed by mathematical modeling. The EIT can be used to visualize changes in blood flow and volume known to occur during evoked activity in the brain, in a manner similar to functional MRI (fMRI). It can also be used as a low-cost portable imaging system for cognitive neuroscience studies at the bedside or in psychological laboratories. EIT can project a deep image into the head, which would allow a much more accurate diagnosis of epilepsy syndromes. EIT can also detect impedance changes in the brain due to increased blood flow or swelling of cells during epilepsy. In the pre-surgical assessment for refractory epilepsy, EIT images can be acquired and recorded continuously for days. This makes it possible to image the affected area and thus avoid intracranial electrodes and their associated morbidity. EIT could provide a routine bedside method for continuous image production, which can identify the site of onset of seizures in patients awaiting surgery to remove the epileptic trigger zone [69]. The treatment of strokes is based on the use of thrombolytic agents, such as tissue plasminogen activator (tPA), which allow a significant reduction in morbidity if they are administered a few hours after their onset. However, it is essential that neuroimaging be performed prior to administration, as tPA is only safe if given in the event of stroke due to infarction; if hemorrhage exists, the thrombolytic effect may prolong the damage. However, the appropriate neuroimaging devices are not always available. The EIT could be used to provide rapid and convenient neuroimaging for this purpose. It is inexpensive, small, portable, and secure, and could be made available to emergency departments for screening prior to administration of tPA [70].

*Rapid changes* activities occur over tens of milliseconds due to the opening of ion channels and the flow of ions. These rapid changes are imaged in the brain, treating it as a network of resistances. The EIT can be used to picture the rapid changes over milliseconds that occur during electrical activity in the brain. This is technically very demanding, as the changes are rapid and at the limits of detectability. However, such imaging is currently not possible by any other method. Possible work aims that the EIT could be used to produce images during repeated responses with a temporal resolution of a few milliseconds and a spatial resolution of a few cubic millimeters and allow the quantitative analysis and mathematical modeling of the activity evoked in neuro-anatomical circuits. Several research projects are dedicated to studying the feasibility of imaging brain activity using EIT. Some have looked at EIT repetitive visual stimulation [71]. It was performed in healthy adult volunteers. Measurements were made with 32 EEG Ag/AgCl cup electrodes at a frequency of 1 Hz, observed with light-emitting diode glasses over the entire visual field of the subject. The EIT was recorded with 30 combinations of injection electrodes in pairs, using a current with a frequency of 2.5 kHz and amplitude of 350 μA. The current electrodes were opposed to each other across the head to maximize the sensitivity of the EIT. The reconstructed images demonstrated that the localization accuracy was < 3 mm at all sites. Further work aims to investigate the possibility of using EIT to visualize rapid neuronal activity during somatosensory responses evoked in the brain of anesthetized subjects [63]. It has been shown that it is possible to image impedance changes in the cortex but not impedance changes in the thalamus.

Recent research has focused on 3D EIT in order to improve the surgical results in patients with focal epilepsy, thanks to the localization of the foci of the seizures [69]. A recent modeling study demonstrated that simulated activity of the cerebral hippocampus can be visualized with a localization accuracy of 0.5 mm using an intracranial planar array placed on the rat cortex [70]. The series of impedance measurements are transformed into a tomographic image using methods similar to back projection in X-ray tomography. Currently most systems use a "sensitivity matrix". This matrix links the resistivity of each pixel in the images, to the voltage measurements.

Initially, the reconstruction is performed with the assumption that the subject was a two-dimensional circle. In practice, this is true for small impedance changes of less than 20%. However, this is not true for larger changes, and in this case a non-linear approach with a logic loop can be used. Precise images can be produced, typically with a spatial resolution of about 10% of the subject's diameter.

Several research groups have focused on the study of brain activity monitoring. It is understood that certain pathologies of the brain, such as ischemia [72] (decrease in arterial blood supply to the brain), or conversely, hemorrhages [73] (flow of blood outside its natural circuit) can be detected by EIT because they cause local variations in blood flow.

Although it is difficult to obtain precise measurements (since the skull acts as an insulating barrier to the applied currents), EIT has great potential for benefits. In particular, the localization of epileptic foci assisted by EIT would increase the success rate of surgeries aimed at eliminating the source of seizures in patients (approximately 30% of the population of epilepsy patients) who do not respond to pharmacological treatment.

### Pulmonary ventilation monitoring

The impedance of the lung tissue varies with the air content. Thus, ventilation and changes in expiratory lung volume that occur in the plane of the electrode cause changes in the voltages measured at the surface of the body. Certain physiological phenomena such as perfusion and pulmonary ventilation as well as the pathological presence of fluids, involve the movement of fluids which alters the conductivity distribution of the thorax. In order to study this phenomenon, it is necessary to measure the effect of these movements on the conductivity distribution, [41, 62, 74, 75].

EIT uses the injection of high frequency, low amplitude electrical currents, through 16 or 32 electrodes placed around the thorax, to obtain images of a cross section of the lungs [76, 77]. These currents pass through the thorax following pathways that vary depending on the shape of the chest wall and the distribution of thoracic impedances. The resulting electrical potentials on the surface of the chest wall are measured and used to obtain the distribution of electrical impedance in the chest. Studies have shown that using 16 and 32 electrodes around the chest provides precise measurements for estimating lung function. It has also been shown that in order to refine the measurements, the estimation of lung function from data obtained from the sixth intercostal space is more reliable than that obtained from the fourth intercostal space. Finally, a seated position is easier to tolerate by the subjects [42].

**Fig. 6:**
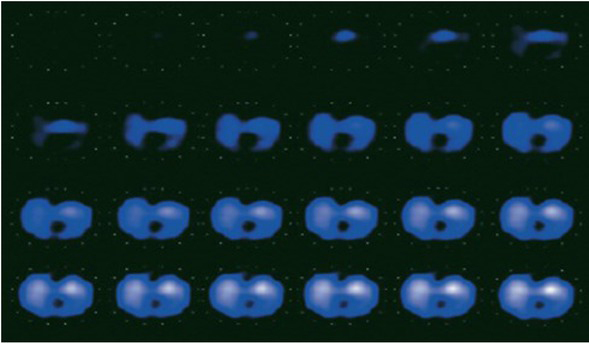
Series of dynamic images showing air filling during inspiration by the PulmoVista500 system [81].

This monitoring offers a response to the needs of clinicians, by making it possible to visualize in real time the variations in thoracic conductivity and to deduce therefrom an index of the variations in the volume of air in each lung. The physiological information provided by the EIT makes it possible to evaluate unilateral lung function, measure pulmonary arterial pressure, monitor the respiratory profile, analyze changes in the shape of the upper respiratory tract, estimate the volumes of chest fluid, study ventilation and perfusion in the region of interest in the thorax [78], adapt ventilator therapy: the settings of ventilators guided by EIT improve gas exchange, respiratory mechanics [79], and to the detection of pneumothorax which is a relatively common complication in intensive care patients undergoing mechanical ventilation or undergoing invasive procedures such as central venous line placement or thoracentesis [77]. Interpretation of EIT images is however difficult due to their low spatial resolution and the superposition of different physiological phenomena [80]. Coronavirus (COVID-19) pandemic and patient monitoring by EIT will be an important topic for the next few years. Fig 6 presents the reconstructed image by the PulmoVista500 system.

### Monitoring of Heart Activity

The conductivity of lungs changes depending on whether they are empty or filled with air. This change in the distribution of thoracic conductivity depends on the cardiac cycle due to cardiac contraction and blood perfusion. Likewise, the heart's conductivity changes throughout the cardiac cycle depending on the amount of blood it contains. It is therefore possible to assess stroke volume (SV) by EIT [82, 83, 84, 85, 86].

**Fig. 7:**
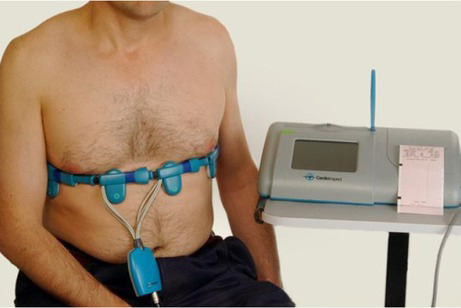
Cardiac monitoring by CardioInspect system [88].

Typically, a single plane of electrodes is used, seeking to reconstruct a cross section of the body. Electrical measurements were taken from electrodes on the surface of the body. There are a few portable EIT systems such as PulmoTrace [87] and CardioInspect [88] that can measure the systolic ejection volume, Fig. 7.

Studies have shown that most image reconstruction algorithms ignore the 3D characteristics of current in the body. In fact, a significant amount of current flows out of the electrode plane, creating distortions in the resulting images. To solve this linearized 3D inverse problem, reconstruction algorithms can be used with multiple electrode planes to reduce distortions of body structures [27, 89].

Recent research has inferred that cardiac EIT is hampered by a limited understanding of the physiological origin of the measured signals. They were therefore based on a 4D thoracic model, and simulations were carried out to give an overview of the genesis of the cardiac EIT signal. The results showed that the heart region is dominated by ventricular activity with only a small contribution (<10%) from the atrium [90], whether the belt is transverse or oblique. Cardiac monitoring by EIT can diagnose several cardiovascular abnormalities: congestive heart failure, hypertension or even coronary artery disease. This technique is also suitable for non-invasive cardiac stroke volume measurement.

### Cancer

As early as 1926, Fricke and Morse reported that tumor tissue exhibited different electrical properties from healthy tissue [91]. Additional studies have also suggested that a reliable diagnosis can be based on these differences. Thus, knowledge of the electrical conductivity of tissues is necessary and can be used for the detection of tumors through the measurement of bioimpedance

For example, Haemmerich [92] has shown that at 1 MHz, healthy tissue has an electrical conductivity of (4.61 ± 0.42) 10^-1^ S/m while hepatic tumors have an electrical conductivity of (2.69 ± 0.91). This shows that the two types of tissue can be differentiated by their electrical conductivity. Measuring at different frequencies also makes it possible to distinguish between normal and cancerous tissues, for example in the breast, for the skin or for the prostate [93, 94].

The impedance of malignant cells has been shown to differ from that of healthy cells. Thus, as the electrical properties of cancerous and non-cancerous lesions differ depending on the frequency, the conductivity images obtained by the EIT would allow them to be objectively discriminated, thus ensuring an early diagnosis of cancer.

For the skin cancer, the measuring system includes a probe which incorporates an array of planar electrodes which is disposable for hygienic reasons (16 to 64 electrodes). To accommodate different lesion sizes and provide multiple resolutions, the system also supports electrode spacings of 2-4 mm [18]. Reconstruction of skin images allows visualizing lesions of different sizes and at varying depths. Since voltage measurements are acquired around the region of interest and planar electrodes are used, image reconstruction is a poorly conditioned problem. The 3D images obtained using data acquired with a planar electrode topology exhibit several artifacts. This problem can be solved by combining the MAP algorithm with the truncated singular value decomposition. The incidence rate for skin cancer is the highest among all cancers [95]. Several diagnostic techniques have been developed (dermoscopy, optical tomography, ultrasound, etc.) [96], but they are not applicable for routine dermatological examinations. Thus, EIT presents a promising solution to this type of cancer. It is non-invasive, quick and easy to use. In addition, reconstructing images of the skin can identify malignant areas, helping the clinician to choose the optimal region for biopsy. Despite these advantages, no EIT system adapted to the dermatological context has yet been produced.

Breast cancer is currently detected by palpation, mammography, ultrasound, or MRI. Research continues to find ways to diagnose breast cancer more accurately and less invasively, and to allow earlier detection. EIT exploits the conductivity that differs between breast tumors and normal breast tissue [97]. These differences are explained by the structural changes (changes in the volume of electrolytes and cell membranes, tumor vascularization, etc.) that cells undergo when invaded by cancer. These changes affect how the applied alternating current is distributed in the body into conduction current (proportional to conductivity) and displacement current (proportional to relative permittivity and excitation frequency) [65, 68, 93, 98].

The EIT system consists of a hand-held scanning probe and a computer screen that shows two-dimensional images of the breast. An electrode is placed on the patient's arm. A weak electric current is transmitted through the electrode. It passes through the breast where electrical conductivity is measured by the scanning probe placed on the breast. An image is then generated from the electrical impedance measurements and displayed on the computer screen. Other devices use a compact array of 256 electrodes and small electrical pulses (0.5 mA, 50 kHz) for examining the breasts [99, 100]. The EIT-MF allows the potentials resulting from the conduction and displacement currents to be measured at different frequencies and to produce a characteristic impedance of the tissues. Screening for breast cancer by EIT-MF, for a given physiological state, is based on the fact that each tissue has a particular conductivity and permittivity and can help to differentiate this tissue from other tissues and physiological states of the same tissue. The resolution of the resulting image depends on the number of electrodes. Low impedance areas usually appear as more intense (whiter) areas. Breast cancer screening is mainly done by mammography which has a high sensitivity with poor specificity [93]. EIT can be a much more convenient way to screen for breast cancer routinely.

### Other Applications

The EIT allows the measurement of motor activity in the intestine and the assessment of gastric emptying or peristalsis. This is because the change in the electrical conductivity of the stomach is due to the movement of fluids and food through the digestive system. To increase the contrast in conductivity, food is used, generally pieces of meat or mash containing a slight excess of salt [68]. The principle of EIT for gastric emptying is based on the use of 16 electrodes placed circularly in a plane around the abdomen. Alternating current is applied to each pair of adjacent electrodes by rotation and the potential differences between the remaining 13 electrodes are measured. The data collection contains approximately 150 cycles (it is considered that one cycle is performed when a pair of electrodes act as the emitter electrodes). Recent studies have shown that EIT has been shown to be as effective as scintigraphy in assessing gastric emptying in patients undergoing nasogastric liquid nutrition [101]. It measures emptying by monitoring changes in epigastric impedance as a meal gradually empties from the stomach. Thus, the advantage of EIT is that it avoids ingestion of radioisotopes while using much simpler and less expensive instrumentation. Additionally, medical applications include non-intrusive diagnosis of lymph problems and for imaging clots in blood flows [86, 102], and the detection of thrombus in blood flow [68, 103, 104].

Another potential application of recent activity is EIT of bladder size [105] or state detection [106].

## Conclusion

EIT is a promising imaging technique with a great potential and many applications. It is based on the principle that the electrical properties of each biological tissue vary in a specific way. Images are reconstructed from a series of measurements acquired using electrodes placed around the region of interest. The instrumentation and image reconstruction methods and systems were presented. Particularly, linear approach algorithm, simple stage reconstruction, Sheffield back-projection, Newton-Raphson and optimization by particle swarm algorithm were described.

Although EIT is not yet considered a regular medical imaging modality, it offers much potential for rapid tomographic imaging at low cost. This promising technique has several medical applications such as monitoring of brain activity, pulmonary ventilation monitoring, monitoring of heart activity and cancer. A special attention is made to the papers published during the last years (from 2015 until 2020). EIT has generated interest for several reasons. The equipment is inexpensive, easy to use, non-invasive, and obtains data in a way that is comfortable for patients since it does not use radiation or require patient preparation.
